# Supplementary material for: OCT and ERG Techniques in High-Throughput Phenotyping of Mouse Vision
Source: Genes (Basel). 2023 Jan 22;14(2):294. doi: 10.3390/genes14020294 (PMC9956909; doi:10.3390/genes14020294)
Supplement: Supplementary file 1 [file genes-14-00294-s001.zip › genes-2106843-supplementary.pdf]

Supplementary Table S1. List of single-gene knock out mouse lines which underwent vision tests in the Czech Centre for Phenogenomics between years 2018 – 2022 as part of the IMPC phenotyping project. Genes are sorted alphabetically, zygosity is stated as "Hom" homozygous, "Het" heterozygous and "Hem" hemizygous, respectively. The phenotyping of some lines was not yet finished at the time of publishing (marked as "TBC", "to be completed"); meaning that the examination was carried out in less than 14 individuals (7 males + 7 females). In these lines the results should be understood as preliminary and taken with caution. Anterior eye segment was examined by Pentacam Scheimpflug camera (OCULUS Optikgeräte, Germany) and evaluated by an experienced examiner as normal or abnormal with respect to the lens and cornea opacity and their overall morphology. Retinal superficial vasculature and the retina as such were assessed from fundus images and retinal cross sections, respectively, obtained by Spectralis SD-OCT (Heidelberg Engineering, Germany). The examiner evaluated number of vessels and their structure and pattern as normal or abnormal for each eye. Similarly, the retina was classified as normal or abnormal based on its overall morphology, obvious thinning or thickening, or presence of a substantial proportion of dysplasia. The number of normal and abnormal cases of the anterior segment, the retina and its vasculature were statistically compared to wild type values by Fisher's exact test with the threshold p-value set to 0.05. Non-significant results are displayed as "ns". ERG was recorded in selected mouse lines with RETI-animal system (Roland-Consult, Germany). For the purpose of this table the amplitudes of scotopic waves a and b evoked by single flashes of 10 $cd.s.m^{-2}$ luminance were compared to wild type controls by unpaired t-test. Abnormality is reported for $p < 0.05$ obtained in either test. "NA" stands for lines that were not tested in ERG.

Gene	Zygosity	Phenotyping finished	Anterior segment	Blood vessels	Retina	ERG
0610040J01Rik	Hom	Yes	ns	ns	ns	ns
1810011H11Rik	Hom	Yes	Abnormal	ns	ns	NA
1810041L15Rik	Hom	Yes	ns	Abnormal	ns	NA
3425401B19Rik	Hom	Yes	Abnormal	ns	ns	NA
Abca5	Hom	TBC	ns	Abnormal	ns	NA
Abtb1	Hom	Yes	ns	ns	ns	NA
Ace2	Hem	Yes	Abnormal	Abnormal	Abnormal	NA
Adk	Het	Yes	ns	ns	ns	NA
Agpat5	Hom	Yes	ns	ns	ns	NA
Aim2	Hom	Yes	ns	ns	ns	NA
Ak7	Het	Yes	ns	ns	ns	ns
Amelx	Hem	Yes	Abnormal	ns	ns	NA
Ano2	Hom	Yes	ns	ns	ns	NA
Anxa1	Hom	Yes	ns	ns	ns	NA
Apbb1ip	Hom	Yes	ns	ns	ns	ns
Apobec1	Hom	Yes	ns	ns	ns	NA
Arg2	Hom	Yes	ns	ns	ns	ns
Arl16	Hom	Yes	ns	ns	ns	NA
Asb1	Hom	Yes	Abnormal	ns	ns	NA
Asb16	Hom	Yes	Abnormal	ns	ns	NA
Asb4	Hom	Yes	ns	ns	ns	NA
Asb5	Hom	Yes	Abnormal	Abnormal	ns	NA
Asb7	Hom	Yes	Abnormal	ns	ns	NA
Ascl5	Hom	Yes	ns	ns	ns	NA
Asphd1	Hom	Yes	ns	ns	ns	NA
Ate1	Het	TBC	ns	ns	ns	NA
Atf2	Het	Yes	ns	ns	ns	NA
Atg7	Het	Yes	ns	ns	Abnormal	NA
Atp1a1	Het	Yes	ns	ns	ns	NA
Babam1	Hom	Yes	Abnormal	ns	ns	NA
Bbip1	Het	Yes	ns	ns	Abnormal	ns
Bbs12	Het	Yes	Abnormal	ns	ns	NA

Birc6	Het	Yes	ns	ns	Abnormal	NA
Bmp6	Hom	Yes	ns	ns	ns	NA
Brinp3	Hom	Yes	Abnormal	ns	ns	NA
Btbd1	Hom	Yes	ns	ns	ns	NA
Btbd18	Hom	Yes	Abnormal	ns	ns	NA
Btbd7	Hom	Yes	Abnormal	ns	ns	Abnormal
Btbd8	Hom	Yes	ns	Abnormal	ns	NA
Bysl	Het	Yes	ns	ns	ns	NA
Cabin1	Het	Yes	ns	Abnormal	ns	NA
Cactin	Het	Yes	ns	ns	ns	NA
Calb1	Hom	Yes	ns	ns	Abnormal	ns
CamK4	Hom	Yes	ns	ns	ns	NA
Canx	Hom	TBC	ns	ns	ns	NA
Car6	Hom	Yes	Abnormal	ns	Abnormal	Abnormal
Card11	Hom	Yes	Abnormal	ns	ns	NA
Casp14	Hom	Yes	ns	ns	ns	NA
Ccdc110	Hom	TBC	ns	ns	ns	NA
Ccdc198	Hom	Yes	ns	Abnormal	ns	NA
Ccl28	Hom	Yes	ns	ns	ns	NA
Ccr3	Hom	Yes	Abnormal	ns	ns	NA
Cd27	Hom	Yes	ns	Abnormal	ns	ns
Cd38	Hom	Yes	ns	ns	ns	NA
Cer1	Hom	Yes	ns	ns	ns	ns
Chfr	Hom	TBC	ns	ns	ns	NA
Chuk	Het	Yes	ns	ns	ns	NA
Churc1	Hom	Yes	ns	ns	ns	NA
Clnk	Hom	Yes	Abnormal	ns	ns	NA
Clock	Hom	Yes	ns	ns	ns	NA
Cngb3	Hom	TBC	ns	ns	ns	NA
Cnpy2	Hom	Yes	ns	ns	ns	NA
Cnpy4	Hom	Yes	ns	ns	ns	NA
Coa6	Het	Yes	ns	ns	ns	NA
Cobll1	Hom	TBC	ns	ns	ns	NA
Cog5	Het	Yes	ns	ns	ns	NA
Cops8	Het	TBC	ns	ns	ns	NA
Crocc2	Hom	TBC	ns	ns	ns	NA
Crx	Hom	Yes	ns	ns	Abnormal	Abnormal
Cst6	Het	Yes	Abnormal	ns	Abnormal	NA
Cul4a	Hom	Yes	ns	Abnormal	Abnormal	NA
Cul9	Hom	Yes	ns	ns	Abnormal	NA
Cyp39a1	Hom	Yes	ns	Abnormal	ns	NA
Dach2	Hom	TBC	ns	ns	ns	NA
Dazap1	Het	Yes	Abnormal	ns	Abnormal	NA
Dcaf12	Hom	Yes	ns	Abnormal	Abnormal	NA
Dcaf15	Hom	TBC	Abnormal	Abnormal	Abnormal	NA
Dcaf5	Hom	TBC	ns	ns	Abnormal	NA
Dcaf8	Hom	Yes	ns	ns	Abnormal	NA
Ddb1	Het	Yes	ns	ns	ns	NA
Ddi2	Het	Yes	ns	Abnormal	ns	NA
Dtx4	Het	Yes	ns	Abnormal	ns	NA
Emsy	Het	Yes	ns	ns	ns	NA

Fam120c	Hom	Yes	ns	ns	ns	NA
Fam122b	Hem	Yes	ns	ns	ns	ns
Fam124a	Hom	Yes	ns	ns	ns	NA
Fam126a	Hom	Yes	Abnormal	Abnormal	ns	NA
Fam129a	Hom	Yes	ns	ns	ns	NA
Fam160b2	Hom	Yes	ns	ns	ns	NA
Fam161b	Hom	Yes	ns	ns	ns	NA
Fam167a	Hom	Yes	ns	Abnormal	ns	ns
Fam185a	Hom	Yes	ns	ns	ns	NA
Fam205c	Hom	Yes	ns	ns	ns	NA
Fam208a	Het	Yes	ns	ns	ns	NA
Fam219a	Hom	Yes	ns	ns	ns	NA
Fam221a	Hom	Yes	ns	ns	ns	NA
Fam227b	Hom	TBC	ns	ns	ns	NA
Fam45a	Hom	Yes	ns	ns	ns	NA
Fam46a	Hom	Yes	Abnormal	ns	ns	NA
Fam53a	Hom	Yes	ns	ns	ns	NA
Fam60a	Het	Yes	ns	ns	ns	NA
Fam71d	Hom	Yes	ns	ns	ns	NA
Fam71f1	Hom	Yes	ns	ns	ns	NA
Fam71f2	Hom	Yes	ns	ns	ns	NA
Fam81a	Hom	Yes	ns	ns	ns	NA
Fam83d	Hom	Yes	ns	ns	ns	NA
Fam83h	Hom	Yes	ns	ns	ns	NA
Fam84b	Hom	Yes	ns	ns	Abnormal	Abnormal
Fam96b	Het	Yes	ns	ns	Abnormal	NA
Fat3	Hom	TBC	ns	ns	ns	NA
Fbxl3	Hom	Yes	ns	ns	Abnormal	Abnormal
Fbxo25	Hom	Yes	ns	ns	ns	NA
Fbxw15	Hom	Yes	ns	ns	ns	NA
Fbxw16	Het	Yes	ns	ns	ns	NA
Fbxw18	Het	Yes	Abnormal	Abnormal	ns	NA
Fbxw20	Hom	Yes	ns	ns	ns	NA
Fbxw25	Hom	TBC	ns	ns	ns	NA
Fgd3	Hom	Yes	ns	ns	ns	NA
Fgf14	Hom	Yes	ns	ns	ns	NA
Fgf20	Hom	Yes	Abnormal	ns	ns	NA
Flt3	Hom	Yes	ns	ns	ns	NA
Fnip1	Hom	TBC	ns	ns	ns	NA
Gabra5	Hom	Yes	ns	ns	ns	NA
Gak	Het	TBC	ns	ns	ns	NA
Gdf6	Het	Yes	ns	Abnormal	Abnormal	Abnormal
Gm10851	Hom	Yes	ns	ns	ns	NA
Gm20219	Hom	TBC	ns	ns	ns	NA
Gm5148	Het	Yes	ns	ns	ns	NA
Gpatch4	Hom	TBC	ns	ns	ns	NA
Gpr109	Hom	Yes	ns	ns	ns	NA
Gpr81	Hom	Yes	ns	ns	ns	NA
Gpr83	Hom	Yes	ns	ns	ns	ns
Gulo	Hom	TBC	ns	ns	Abnormal	NA
Hcls1	Hom	TBC	ns	ns	ns	NA

Hdac4	Het	Yes	ns	Abnormal	ns	NA
Hormad2	Hom	TBC	ns	ns	ns	NA
Impact	Hom	TBC	ns	ns	ns	NA
Insig1	Hom	Yes	Abnormal	ns	Abnormal	NA
Irak4	Hom	TBC	ns	ns	ns	NA
Itgb6	Hom	Yes	ns	ns	ns	NA
Kbtbd11	Hom	Yes	ns	ns	ns	NA
Kbtbd3	Hom	Yes	ns	ns	ns	NA
Kcnn4	Hom	Yes	ns	ns	ns	NA
Kif16b	Hom	Yes	ns	ns	ns	NA
Kif6	Hom	Yes	ns	ns	ns	NA
Klhl1	Hom	Yes	ns	ns	ns	NA
Klhl6	Hom	TBC	ns	ns	ns	NA
Klk11	Hom	Yes	ns	ns	ns	NA
Klk12	Hom	Yes	ns	ns	ns	NA
Klk13	Hom	Yes	ns	Abnormal	Abnormal	NA
Klk15	Hom	Yes	ns	ns	ns	NA
Klk8	Hom	Yes	Abnormal	ns	ns	NA
Krt1	Hom	TBC	ns	ns	ns	NA
Krt27	Hom	Yes	ns	ns	ns	NA
Krt28	Hom	Yes	ns	ns	ns	NA
Krt33a	Hom	Yes	ns	ns	ns	NA
Krt33b	Hom	Yes	ns	ns	ns	NA
Krt34	Hom	TBC	Abnormal	ns	ns	NA
Krt72	Hom	Yes	ns	ns	ns	NA
Krtap7	Hom	Yes	Abnormal	ns	ns	NA
Krtap8-1	Hom	Yes	ns	ns	Abnormal	NA
Krtap9-5	Hom	Yes	ns	ns	ns	NA
Lamtor4	Het	Yes	Abnormal	ns	Abnormal	NA
Lgr6	Hom	Yes	ns	ns	ns	NA
Lhx5	Het	Yes	ns	ns	ns	NA
Lrig3	Hom	TBC	ns	ns	ns	NA
Lrrc17	Hom	Yes	ns	ns	ns	NA
Ltn1	Hom	Yes	Abnormal	Abnormal	ns	NA
Mapkapk3	Hom	Yes	ns	ns	ns	NA
Marchf3	Hom	Yes	ns	Abnormal	ns	NA
Marveld3	Hom	Yes	ns	Abnormal	ns	NA
Meis3	Hom	Yes	ns	ns	ns	NA
Mex3b	Hom	Yes	ns	ns	ns	NA
Mfng	Hom	Yes	Abnormal	ns	ns	ns
Mmp20	Hom	Yes	ns	ns	ns	NA
Mpp1	Hom	TBC	Abnormal	ns	ns	NA
Myo1g	Hom	Yes	ns	ns	ns	NA
Naa38	Het	Yes	Abnormal	ns	ns	NA
Nfam1	Hom	Yes	ns	ns	ns	NA
Nfe2l2	Hom	TBC	ns	ns	ns	NA
Nmi	Hom	TBC	ns	ns	ns	NA
Nsmce1	Het	Yes	ns	Abnormal	Abnormal	NA
Nuggc	Hom	Yes	ns	ns	ns	ns
Nwd2	Hom	Yes	ns	Abnormal	ns	NA
Nxnl2	Het	TBC	ns	ns	ns	NA

Oas1c	Hom	Yes	ns	ns	ns	NA
Odaph	Hom	Yes	ns	Abnormal	ns	NA
Odf3b	Hom	Yes	ns	ns	ns	NA
Otop1	Hom	TBC	ns	ns	ns	NA
Pank2	Hom	TBC	ns	ns	ns	NA
Papd7	Hom	Yes	ns	ns	ns	NA
Pcid2	Het	TBC	ns	ns	ns	NA
Pdgfd	Hom	Yes	ns	Abnormal	ns	NA
Pdgfra	Het	TBC	ns	Abnormal	ns	NA
Pgc	Hom	TBC	ns	ns	ns	NA
Pglyrp4	Hom	Yes	ns	ns	ns	NA
Pknox2	Hom	Yes	ns	Abnormal	Abnormal	NA
Pla2g2f	Hom	Yes	ns	Abnormal	ns	NA
Prdm8	Hom	Yes	Abnormal	Abnormal	Abnormal	Abnormal
Prg4	Hom	Yes	ns	ns	ns	NA
Proser2	Hom	Yes	ns	ns	ns	NA
Prss12	Hom	Yes	Abnormal	ns	ns	NA
Prss21	Het	Yes	ns	ns	ns	NA
Prss33	Hom	Yes	ns	ns	ns	NA
Prss35	Hom	Yes	ns	ns	ns	NA
Prss47	Hom	Yes	ns	Abnormal	ns	NA
Prss48	Hom	Yes	ns	ns	ns	ns
Prss54	Hom	Yes	ns	ns	ns	NA
Prss55	Hom	Yes	ns	Abnormal	ns	NA
Prss57	Hom	Yes	ns	ns	ns	NA
Psen2	Hom	Yes	ns	Abnormal	Abnormal	NA
Psmb11	Hom	Yes	ns	ns	ns	NA
Pstpip1	Hom	Yes	ns	ns	ns	NA
Ptchd4	Hom	Yes	ns	ns	ns	NA
Rasgrp3	Hom	Yes	ns	ns	Abnormal	NA
Rffl	Hom	Yes	ns	Abnormal	Abnormal	NA
Rhag	Hom	Yes	ns	ns	ns	NA
Rnf128	Hem	Yes	ns	ns	ns	NA
Rnf14	Hom	Yes	Abnormal	ns	ns	NA
Rnf148	Hom	Yes	ns	ns	ns	NA
Rnf19b	Hom	Yes	Abnormal	ns	Abnormal	NA
Rnf208	Hom	Yes	ns	ns	ns	NA
Rnf223	Hom	Yes	Abnormal	ns	Abnormal	NA
Rnf34	Hom	TBC	ns	ns	ns	NA
Rnf4	Het	Yes	ns	Abnormal	ns	NA
Rnf5	Het	Yes	ns	ns	ns	NA
Rnft1	Hom	Yes	ns	ns	Abnormal	NA
Ror1	Het	Yes	ns	Abnormal	ns	NA
Rras2	Hom	Yes	ns	ns	ns	NA
Sall3	Het	Yes	Abnormal	ns	ns	NA
Sbsn	Hom	Yes	ns	ns	ns	NA
Sbspon	Hom	Yes	ns	ns	ns	NA
Scel	Hom	Yes	Abnormal	ns	ns	NA
Sla2	Hom	Yes	ns	ns	ns	NA
Slc15a1	Hom	TBC	ns	ns	ns	NA
Slc38a7	Hom	TBC	Abnormal	ns	ns	ns

Slc38a8	Hom	Yes	ns	ns	ns	ns
Sox14	Hom	Yes	ns	ns	ns	NA
Spink13	Hom	Yes	Abnormal	Abnormal	ns	NA
Spred1	Hom	Yes	Abnormal	ns	Abnormal	ns
Spryd4	Hom	Yes	ns	Abnormal	Abnormal	NA
Strip2	Hom	Yes	ns	ns	Abnormal	NA
Sult1a1	Hom	Yes	ns	ns	ns	NA
Syngr1	Hom	TBC	Abnormal	ns	ns	NA
Tcaf2	Hom	TBC	ns	ns	ns	NA
Tekt1	Hom	TBC	ns	ns	ns	NA
Thoc1	Het	Yes	ns	ns	ns	NA
Tkfc	Hom	TBC	Abnormal	ns	ns	NA
Tmem240	Hom	Yes	ns	ns	ns	NA
Tmem47	Hem	Yes	ns	ns	ns	NA
Tmem60	Hom	Yes	ns	ns	Abnormal	NA
Tmem62	Hom	Yes	ns	ns	ns	NA
Tnfr60	Hom	Yes	ns	ns	ns	ns
Tomm6	Het	Yes	ns	ns	ns	NA
Tppp3	Het	TBC	ns	ns	Abnormal	NA
Trim15	Hom	Yes	ns	ns	ns	NA
Tyro3	Hom	Yes	ns	ns	ns	NA
Ubd	Hom	Yes	Abnormal	ns	ns	NA
Ube2n	Het	Yes	ns	ns	ns	NA
Ubl4b	Hom	Yes	Abnormal	ns	ns	NA
Uchl4	Hom	Yes	ns	ns	ns	NA
Uox	Het	TBC	ns	ns	ns	NA
Vamp4	Hom	TBC	ns	ns	Abnormal	NA
Vegfd	Hom	Yes	Abnormal	ns	ns	NA
Vnn3	Hom	TBC	Abnormal	ns	ns	NA
Wdr49	Hom	Yes	ns	ns	ns	NA
Wdr63	Hom	TBC	ns	ns	ns	NA
Wiz	Het	Yes	ns	ns	Abnormal	ns
Xrcc5	Hom	TBC	ns	Abnormal	Abnormal	Abnormal
Zc2HC1a	Hom	Yes	ns	ns	ns	NA
Zc2Hc1b	Hom	TBC	Abnormal	ns	ns	NA
Zc2HC1c	Hom	Yes	ns	ns	ns	NA
Zc3h8	Het	Yes	ns	ns	ns	ns
Zfat	Het	Yes	ns	ns	ns	NA
Zfp474	Hom	Yes	Abnormal	ns	ns	NA
Zfp644	Hom	Yes	ns	ns	ns	NA
